# How Can We Improve Outcomes for Patients and Families Under Palliative Care? Implementing Clinical Audit for Quality Improvement in Resource Limited Settings

**DOI:** 10.4103/0973-1075.63128

**Published:** 2010

**Authors:** Lucy Selman, Richard Harding

**Affiliations:** King’s College London, Department of Palliative Care, Policy and Rehabilitation, Cicely Saunders Institute, Bessemer Road, Denmark Hill, London SE5 9PJ, UK

**Keywords:** Audit, Outcomes, Quality improvement, Quality of care

## Abstract

Palliative care in India has made enormous advances in providing better care for patients and families living with progressive disease, and many clinical services are well placed to begin quality improvement initiatives, including clinical audit. Clinical audit is recognized globally to be essential in all healthcare, as a way of monitoring and improving quality of care. However, it is not common in developing country settings, including India. Clinical audit is a cyclical activity involving: identification of areas of care in need of improvement, through data collection and analysis utilizing an appropriate questionnaire; setting measurable quality of care targets in specific areas; designing and implementing service improvement strategies; and then re-evaluating quality of care to assess progress towards meeting the targets. Outcome measurement is an important component of clinical audit that has additional advantages; for example, establishing an evidence base for the effectiveness of services. In resource limited contexts, outcome measurement in clinical audit is particularly important as it enables service development to be evidence-based and ensures resources are allocated effectively. Key success factors in conducting clinical audit are identified (shared ownership, training, managerial support, inclusion of all members of staff and a positive approach). The choice of outcome measurement tool is discussed, including the need for a culturally appropriate and validated measure which is brief and simple enough to incorporate into clinical practice and reflects the holistic nature of palliative care. Support for clinical audit is needed at a national level, and development and validation of an outcome measurement tool in the Indian context is a crucial next step.

## INTRODUCTION

Palliative care in resource-poor settings has made enormous advances in providing better care for patients and families living with progressive disease. Now that innovative, sustainable palliative care facilitates are in place in India, these clinical services are well placed to begin considering how to implement simple ways to strive for quality improvement. Clinical audit* is recognized globally to be essential in all healthcare, enabling quality of care to be monitored and improved. While clinical audit is common within palliative care in the developed world, it is far less common in developing country settings, including India.[1]

In this article we outline some of the reasons why clinical audit is so important in palliative care, and make suggestions for how Indian palliative care services can successfully engage with the audit process. Many of our suggestions come out of experience managing the Encompass study (2006-2008), during which the first clinical audit of palliative care services in sub-Saharan Africa was conducted. Working with principal investigators and local research nurses at four palliative care services in South Africa and one in Uganda, we developed a model for palliative care clinical audit in developing country settings which may be relevant to the Indian setting.

Here, we aim to introduce the concept of clinical audit within the palliative care setting, and share some of the lessons learnt during that project.

## MEASURING QUALITY IN HEALTHCARE

**Figure 1:**
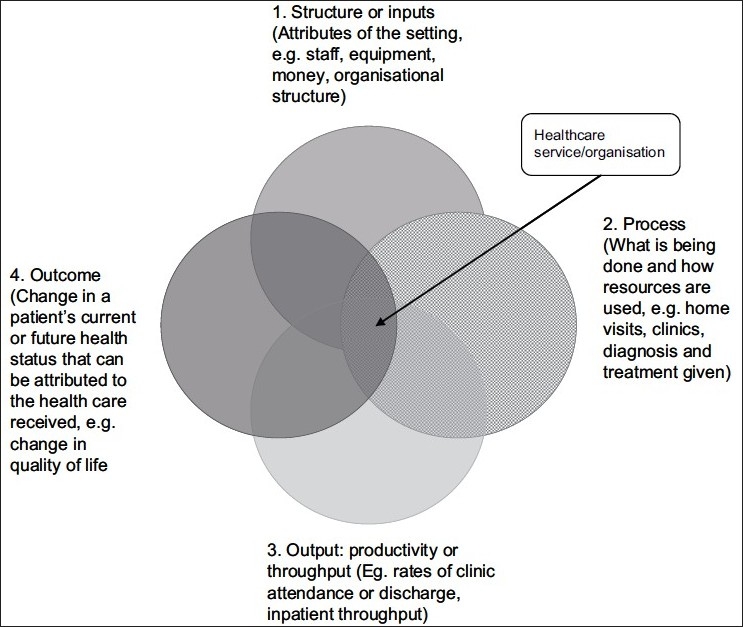
Contributors to the quality of a palliative care organization (adapted from[13])

The quality of a healthcare system (or organization) relates to how effective that system or organization is in achieving it aims. The quality of an organization can be represented and assessed using a four-part model of structure, process, output and outcome [Figure 1]. Each of the four aspects of quality assessment interact, e.g. good structure increases the likelihood of good process, and good process increases the likelihood of good outcome.[2]

It can be useful to collect and reflect on process and output data, e.g. the time between referral and a patient being seen, or the number of referrals to a service, in order to demonstrate or understand the demand for services and practical issues of meeting that demand. However, while assessing how many home care visits are made on each day, or how many patients are seen in a month may provide useful information about how a service is run, does not tell us anything about patients’ or family members’ experience of care (for example, having received home care, did the patient’s pain improve?). Only assessing outcomes of care can provide this kind of information. The outcomes of care (i.e. (4.) in the model) are therefore of particular relevance in measuring quality, as they measure directly the relevance of care for patients, families and society as a whole.

## WHAT IS CLINICAL AUDIT?

Clinical audit is one way to measure quality in healthcare. It can be defined as the ‘systematic critical analysis of the quality of clinical care including procedures used for diagnosis and treatment, the use of resources and resulting outcome and quality of life.’[3] In other words, clinical audit means a) looking at how well we currently perform something (e.g. pain management or psychological support), b) setting a target for how well we want to do it, c) deciding how we will make the improvement, d) putting this in place, and e) measuring again to see if we have achieved the target (see Box 1 for more details). Clinical audit is a cyclical activity, although this is often misunderstood: evaluation on its own is not audit, as the data collected are not used being used to inform changes in service provision which are implemented and evaluated. As Stephen Connor has said, ’Quality assessment must be tied to quality improvement’.[4] For service providers, this could mean conducting a clinical audit every year to make sure the service is always working to improve the care that is delivered. Clinical audit thusbecomes a process of continuous improvement in the quality of care provided by a service, embedded within routine clinical practice and helping to bring about change for the better in terms of patient and family care. Because of its cyclical nature and the dynamism it brings to a healthcare organization, clinical audit has been described as its ‘vital signs’ or ‘pulse’, evidence that the organization is living rather than stagnating.[5]

**Box 1:**
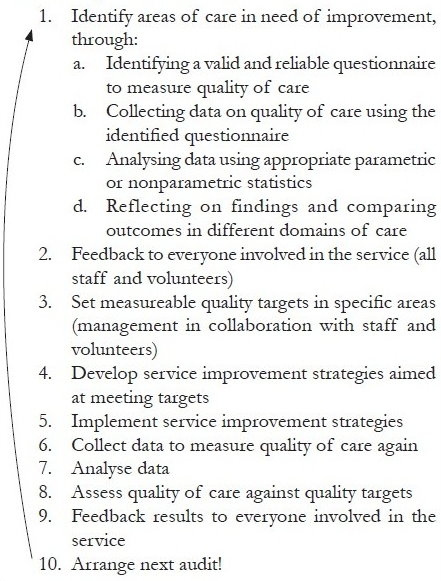
Steps in clinical audit

Importantly, clinical audit is not a process of comparing one service with another and finding one or the other to be lacking in some way. Services will have different aims and philosophies, and, as Stjernswald says, ‘You should not compare chocolate with mango’.[6] Clinical audit is ultimately about service providers being aware of the quality of their own service’s care, including areas for potential improvement, and putting steps in place accordingly. The targets for improvement that services set themselves should reflect their stated aims as well as the areas of care currently requiring extra attention. Only when services are sufficiently similar in terms of their aims and the population cared for is comparison meaningful. In this instance, collecting data for audit does enable the effects of implementing different service models to be better understood. However, actual services will always be more complex than the service models they utilize, and should be considered in the context of the specific characteristics of the communities they serve. Clinical audit should not therefore be seen as a threat, but rather as a facilitator that enables more accurate reflection on service provision.

## OUTCOMES IN PALLIATIVE CARE

Outcomes can be understood as any end result that is attributable to health service intervention,[7] where health is defined as a state of complete physical, mental (which may include spiritual) and social well-being and not merely the absence of disease or infirmity.[8] Clearly, this fits in well with the model of palliative care. In a general healthcare context, the outcome of primary interest is often morbidity; e.g. how many patients died from having an operation of type X at hospital Y in one year. Within palliative care, this aim is less relevant, as the focus of care shifts from extending life to improving the quality of life. A range of outcomes of relevance to palliative care arise out of the holistic aims of palliative care as stated, for example, by the World Health Organization (WHO)[9] (Box 2, also see[10]). Any of these outcomes would be an appropriate focus for measurement and improvement in a clinical audit, depending on the stated aims and priorities of the service.

**Box 2:**
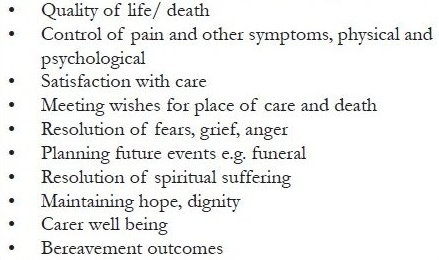
Examples of relevant outcomes in palliative care

## WHY SHOULD SPECIALIST PALLIATIVE CARE MEASURE OUTCOMES OF CARE?

Measuring the outcomes of palliative care has five main benefits. Firstly, it enables improvement of patient and family care on a holistic and individual basis. By obtaining more detailed information about the patient and family by using formal assessment methods in day-to-day practice, healthcare providers are able to tailor and improve their care on a case-by-case basis.

Secondly, assessing the outcomes of care in a formal way enables evidence to be gathered on the impact of care on the patient and family and the effectiveness of the service at meeting its aims. As palliative care is a relatively new specialty, it has much to prove! If systematically collected data is aggregated, analyzed and reviewed, it can be used as evidence of, for example, efficacy or cost-effectiveness. Through measuring the outcomes of care, studies in the US and UK have shown that palliative care improves quality of life, physical well being and symptoms including pain, spiritual well being and psychological well being.[11–13] Such evidence of effectiveness can be used to justify the continuation or expansion of services and secure resources for future services, e.g. by convincing potential funders.[14]

Thirdly, and most crucially in the context of this article, measuring outcomes is fundamental to clinical audit, enabling quality of care to be assessed and improved. Routine collection of data on the outcomes of care in diverse domains enables potential areas for improvement to be identified when the data are reviewed. Service managers can then utilize this data to improve practice, decide where resources should be focused, and set locally-relevant targets for quality of care for the future. Through ongoing audit the achievement of these targets can be monitored, and effective techniques to improve care can be shared with other services.

Fourthly, at the national level, measuring outcomes across a range of services builds an evidence base for setting quality standards and quality indicators appropriate and feasible for different types of service across India. Quality indicators (also called quality markers) are explicitly defined and measurable items referring to the outcomes, processes, or structure of care.[15] As quality indicators are adopted voluntarily, they offer a framework for a palliative care organization to define and track its progress against its own action plans.[16] In India, where quality indicators have not yet been set and service evaluation is at an embryonic stage, there is the chance to learn from omissions in developed countries,[17,18] and ensure that cultural and spiritual aspects of palliative care, and the needs of family carers, are taken into account in national guidance and audit. Ultimately, national standards and quality indicators also need to be subjected to testing through well-designed trials.[19]

Finally, the most important reason to do audit is that patient and family have a right to quality care, matter where they receive care, how that care is delivered, or who delivers the care. Whether a patient is receiving care in a hospital or at home, from trained community volunteers or from medical personnel, the quality of care should be assessed, and the service provider should be committed to its improvement.

## CLINICAL AUDIT IN INDIAN PALLIATIVE CARE

There has been some debate regarding the utility of conducting research, including service evaluation and audit, in developing country settings, where the funds used for such activities would, it is suggested, be better placed in feeding a hungry population.[20] However, in resource-limited contexts it is perhaps even more crucial that available resources, such as staff time and available funds, are used effectively, and that service development is evidence-based.[21–23] As Higginson and Bruera state, measurement and clinical audit are one way to minimize the risk of failure, learn at an early stage about potential problems, and identify successful strategies.[21] Without auditing the outcomes of care, some important domains of palliative care may be neglected. Costs of not conducting clinical audit include providing extra, inappropriate treatment, which wastes patients‘ and families’ time as well as staff time and resources; providing underutilized or inappropriate services; uncontrolled symptoms which are distressing for patients and families, and may lead to delayed discharge or preventable emergency admissions; and other unresolved problems that may cause preventable suffering.[3] For example, research indicates that in the US and UK the needs of family members are often unmet.[24–26] Failure to audit family outcomes (such as family worry, confidence in caring for the patient, and adequacy of information received) may mean that they continue to be neglected.[27]

The need for evaluation and monitoring of quality of care in the Indian setting has been recognized by several authors writing in this journal.[1,6,28] Anil Kumar Paleri reports that the Pain and Palliative Care Policy of the Government of Kerala ’favors locally relevant audit and research at various levels for improving the programs and sharing useful experience.[29] Given the recognition of the importance of evaluation, what is now needed is a clear and concrete action plan, with a commitment from the Indian Association of Palliative Care (IAPC) and service providers to create the conditions necessary for clinical audit to be carried out across palliative care services in India. While there are organizational factors which facilitate successful audit [Box 3], an essential first step is the selection of an outcome measurement tool.

**Box 3:**
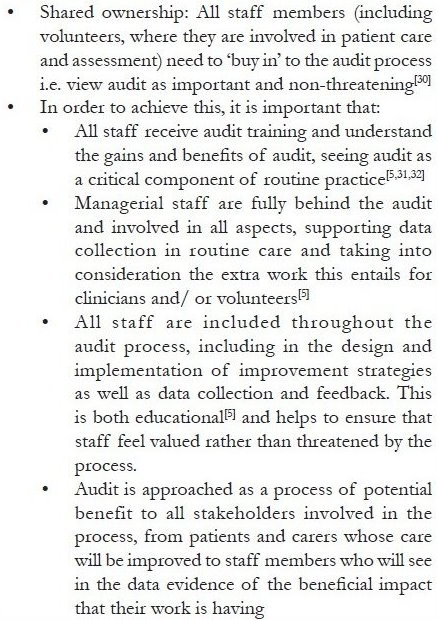
Key factors in conducting a successful audit

## CHOOSING AN OUTCOME MEASUREMENT TOOL

In the UK, a range of measures are used in palliative care service evaluation, the most common being the Support Team Assessment Schedule (STAS).[3,27] However, many palliative care service managers report that they have developed their own assessment tools, or used more informal methods of evaluation such as staff meetings and daily log books.[27] A disadvantage of, using informal methods of assessing outcomes Is that it is difficult to set concrete and meaningful targets which reflect the experience of care by patients and families without inviting them to participate through the use of self-completion (or assisted completion) questionnaires. Using an assessment questionnaire developed in an informal way at your own service is also problematic, as the validity and reliability of the tool is unknown. Established tools used for audit and research purposes have undergone formal psychometric testing to ensure they are valid and reliable, i.e. measure what they set out to measure, and are appropriate in palliative care populations (for example, not too long and burdensome). The validation process aims to identify and eliminate problems in tools, such as systematic bias introduced by wording which leads the respondent to answer one way rather than another, or measurement inadequacies such as floor and ceiling effects. In addition to giving more accurate and valid results, the use of a validated and standardized outcome measurement tool across services means that results from sites with similar service models can be pooled, and results from services or service models with sufficiently similar aims can be meaningfully compared. This can contribute towards the setting of national quality standards, and may also eventually elucidate some of the strengths and weaknesses of specific service models.[4,23]

In Africa, we were able to conduct audit as part of the Encompass project because of careful collaborative science beforehand to develop the APCA African Palliative Outcome Scale (POS).[33,34] The APCA African POS was based on the Palliative Outcome Scale, a tool to assess quality of care that was originally developed and validated in the UK.[35–37] Working with the African Palliative Care Association (APCA) and services across Africa, this outcome measurement tool was developed and validated in a range of different settings, producing a tool that is tailored to and reliable in African palliative care. Services across the continent are therefore able to use the same tool in the knowledge that it is psychometrically valid and reflects their goals of care. Development of a similar tool in India is an essential task.

The use of a questionnaire such as the POS that is specifically designed to measure the quality of palliative care helps to ensure that a wide range of relevant outcomes are assessed.[38] A survey of palliative care services in Britain and Ireland found that although physical aspects of care were audited relatively frequently (by 61% of services), other core aspects of care were rarely audited, including bereavement care (17%), training (13%), and psychological and spiritual care (12%).[27] One of the reasons for this is that the latter domains are considered more difficult to assess formally than physical aspects of care. In the UK survey, 28% of services stated that difficulty of assessment was the reason for not auditing bereavement, 33% gave that response regarding psychological and spiritual care, and 15% regarding training.[27] However, well-validated measures do exist for the assessment of these more intangible concepts, such as quality of life,[39,40] spiritual well being,[41,42] the impact of training,[43,44] and bereavement outcomes.[45,46] As Charlton says, ’Unless these aspects are evaluated regularly, service providers cannot be confident they are successfully achieving their mission to promote optimal palliative care and, where possible, a good death’.[27]

Given the proliferation of palliative care outcome measures in recent years, it would beneficial to build on previous work and revalidate an existing measure in the Indian context. The choice of an appropriate tool would depend on the goals of the IAPC and the properties of the existing tools. However, it is important that the tool chosen for adaption and revalidation meets certain criteria [Box 4].

**Box 4:**
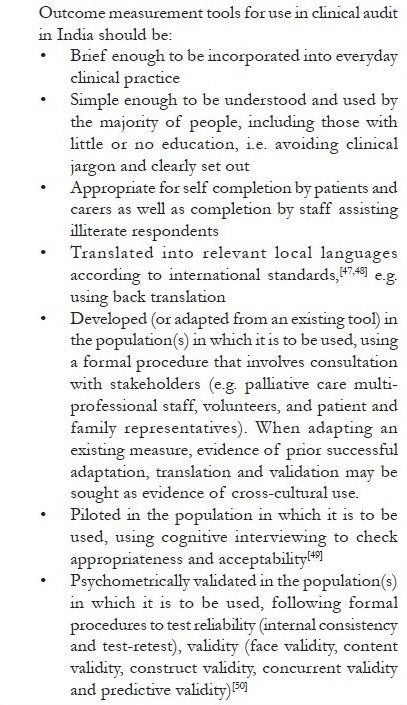
Criteria for the choice of an outcome measurement tool

## CONCLUSION

Collaboration at regional, national and possibly international levels may be required in order to establish the necessary conditions for audit in India. Establishing relevant audit systems will require close interaction between local programs with specific needs and those with audit experience and methodological skills.[21] The development and validation of an Indian palliative care outcome measure will also necessarily be a collaborative process, in order to ensure that the resulting measure is applicable and appropriate across the subcontinent. However, there are also concrete steps that service providers can make in terms of staff education and training about quality improvement, prioritization of research and clinical audit, and collaboration with the IAPC and other services to ensure quality improvement remains high on the national agenda.

The IAPC has an important role to play in fostering increased service evaluation and improvement of existing services,[1] including supporting services conducting audit nationally. As a step towards this, the IAPC and Pallium India are to be congratulated for developing national standards for palliative care, reproduced in the Appendix to this paper. One of the desirable standards is that a palliative care service has a commitment to continuous quality improvement through ongoing use of a standardized audit tool (Point 34[51]). In order to meet this standard, the adaptation and validation of an appropriate outcome measurement tool is an essential next step, as recognized by the Declaration of Venice.[22] Only with such tools can relevant and applicable information regarding the effectiveness of palliative care in India be produced, and evidence-based standards and quality indicators be developed nationally.

**Appendix:** 

**Indian Association for Palliative Care Standards for Palliative Care Providers**			
January 2010			
The Indian Association is planning to initiate a ‘Standards Programme’ for palliative care providers in India. A draft document made by a work group organised by Pallium India Trust, Thiruvananthapuram, was submitted to IAPC. IAPC has taken this up further and following is the final version of the tool which will be launched soon.			
The standards are broadly divided into those that are essential and those that are desirable:			
**Essential standards**			
These essential standards are considered to be the minimum that need to be met for setting up a palliative care service, and all palliative care service providers should try to meet them. This is to ensure that the primary environment for palliative service delivery is made ideal. There can be services which have not met some of these requirements.			
**Desirable standards**			
These are the requirements recommended to strengthen and expand the services. Services may try to achieve the standards mentioned in this section as and when they feel that they are ready for these.			

**Standards**	**Requirement**	**No.**	**Description**

Essential standards	Your hospice/palliative care program has a system in place for whole patient assessment, documentation, and management that includes at minimum	1	Assessment, documentation, and management of pain with at least the body chart and pain scale
		2	Assessment, documentation, and management of other symptoms
		3	Assessment, documentation including family tree, and management of psychosocial issues
		4	Assessment, documentation, and management of spiritual issues
		5	An uninterrupted supply of step 3 opioids to the patient until the end of life
		6	Provision of other essential medications to the patient
		7	A system for documentation of step 3 opioids use including names of patient and identification number, quantity dispensed each time and balance of stock after each transaction
	A palliative service should adopt a team approach. It should have at least	8	A trained doctor with a minimum of 10 days clinical training under supervision
		9	A trained nurse with a minimum of 10 days clinical training under supervision
		10	Team members with skills to deliver psychosocial and spiritual support to the patient and family
	The palliative care service engages the community and does not work in isolation, i.e.	11	There is evidence of involvement with the community in the establishment and ongoing operation of the palliative care service
		12	There is evidence of involvement of other health care professionals in the establishment and ongoing operation of the palliative care service
	The palliative care service supports the health of the team through activities such as	13	Regular monthly palliative care team meetings
	Your hospice/ palliative care program	14	Makes provision for home based care services
		15	Provides bereavement follow up with families
Desirable standards	Your hospice/ palliative care program has	16	Sufficient access to free essential palliative drugs for poor patients
		17	Team members with skills to deliver physical rehabilitation support
		18	The palliative care service has significant contributions from volunteers
		19	An ethical framework to guide palliative care decisions is in place and utilized
		20	The government is supportive of palliative care
		21	Media that are supportive of palliative care work
		22	Other health care professionals that are supportive of palliative care work
	The palliative care service fosters a healthy organizational culture which includes	23	Self-care training
		24	Conflict resolution
		25	Staff stress management
		26	Administrators are supportive of palliative care
		27	Sufficient funds for all current programs
		28	Access to funds for future expansion programs
	A palliative care service has in place a program of education and training that includes	29	Ongoing continuing professional development for the palliative care team
		30	Education programs on palliative care for fellow professionals
		31	Education programs on palliative care for medical/ nursing students
		32	Education programs on palliative care for volunteers
		33	Awareness programs on palliative care for the public
	The palliative care service has a commitment to continuous quality improvement through	34	Ongoing use of a standardised audit tool
		35	Regular clinical discussions
		36	Participation in research

## Footnotes

* Clinical audit in this sense refers to clinical practice improvement. The broad conceptualization of clinical audit used throughout this article is common in the UK, and should be distinguished from narrow understandings of audit as involving a single method of or approach to data collection (e.g. medical note audit).
